# Insights from patients screened but not randomised in the HYPERION trial

**DOI:** 10.1186/s13613-021-00947-w

**Published:** 2021-11-14

**Authors:** J. B. Lascarrou, Gregoire Muller, Jean-Pierre Quenot, Nicolas Massart, Mickael Landais, Pierre Asfar, Jean-Pierre Frat, Jean-Charles Chakarian, Michel Sirodot, Bruno Francois, Guillaume Grillet, Sylvie Vimeux, Arnaud Delahaye, Stéphane Legriel, Didier Thevenin, Jean Reignier, Gwenhael Colin, for the AfterROSC Network

**Affiliations:** 1 Medical Intensive Care Unit, Service de Médecine Intensive Réanimation, University Hospital Center, 30 Boulevard Jean Monnet, 44093 Paris, France; 2 Paris Cardiovascular Research Center, INSERM U970, Paris, France; 3 AfterROSC Network, Paris, France; 4 Medical Intensive Care Unit, Regional Hospital Center, Orleans, France; 5 Department of Intensive Care, Burgundy University Hospital, Dijon, France; 6 Lipness Team, INSERM Research Center LNC-UMR1231, Dijon, France; 7 LabEx LipSTIC, University of Burgundy, Dijon, France; 8 INSERM CIC 1432, Clinical Epidemiology, University of Burgundy, Dijon, France; 9 Medical-Surgical Intensive Care Unit, General Hospital Center, Saint Brieuc, France; 10 Medical-Surgical Intensive Care Unit, General Hospital Center, Le Mans, France; 11 Medical Intensive Care Unit, University Hospital Center, Angers, France; 12 Medical Intensive Care Unit, University Hospital Center, Poitiers, France; 13 INSERM, CIC-1402, équipe ALIVE, Poitiers, France; 14 Poitiers School of Medicine and Pharmacy, Poitiers University, Poitiers, France; 15 Medical-Surgical Intensive Care Unit, General Hospital Center, Roanne, France; 16 Medical-Surgical Intensive Care Unit, General Hospital Center, Annecy, France; 17 Medical-Surgical Intensive Care Unit, University Hospital Center, Limoges, France; 18 INSERM CIC 1435 & UMR 1092, University Hospital Center, Limoges, France; 19 Medical-Surgical Intensive Care Unit, General Hospital Center, Lorient, France; 20 Medical-Surgical Intensive Care Unit, General Hospital Center, Montauban, France; 21 Medical-Surgical Intensive Care Unit, General Hospital Center, Rodez, France; 22 Medical-Surgical Intensive Care Unit, Versailles Hospital, Versailles, France; 23 Medical-Surgical Intensive Care Unit, General Hospital Center, Lens, France; 24 Medical-Surgical Intensive Care Unit, District Hospital Center, La Roche-sur-Yon, France

**Keywords:** Cardiac arrest, Targeted temperature management, Therapeutic hypothermia

## Abstract

**Background:**

Few data are available about outcomes of patients screened for, but not enrolled in, randomised clinical trials.

**Methods:**

We retrospectively reviewed patients who had non-inclusion criteria for the HYPERION trial comparing 33 °C to 37 °C in patients comatose after cardiac arrest in non-shockable rhythm, due to any cause. A good neurological outcome was defined as a day-90 Cerebral Performance Category score of 1 or 2.

**Results:**

Of the 1144 patients with non-inclusion criteria, 1130 had day-90 information and, among these, 158 (14%) had good functional outcomes, compared to 7.9% overall in the HYPERION trial (10.2% with and 5.7% without hypothermia). Considerable centre-to-centre variability was found in the proportion of non-included patients who received hypothermia (0% to 83.8%) and who had good day-90 functional outcomes (0% to 31.3%). The proportion of patients with a good day-90 functional outcome was significantly higher with than without hypothermia (18.5% vs. 11.9%, *P* = 0.003).

**Conclusion:**

Our finding of better functional outcomes without than with inclusion in the HYPERION trial, despite most non-inclusion criteria being of adverse prognostic significance (e.g., long no-flow and low-flow times and haemodynamic instability), raises important questions about the choice of patient selection criteria and the applicability of trial results to everyday practice. At present, reserving hypothermia for patients without predictors of poor prognosis seems open to criticism.

**Supplementary Information:**

The online version contains supplementary material available at 10.1186/s13613-021-00947-w.

## Introduction

The 2021 International Liaison Committee on Resuscitation (ILCOR) guidelines recommend hypothermia at 32–36 °C in patients who are comatose after cardiac arrest [1]. We reported that hypothermia at 33 °C improved day-90 functional outcomes compared to maintaining 37 °C in patients with cardiac arrest in non-shockable rhythm [2]. More recently, a randomised controlled trial (RCT) found that 33 °C did not decrease 6-month mortality compared to normothermia with early treatment of fever after cardiac arrest from cardiac causes [3]. In our trial [2], the large proportion of screened patients who had non-inclusion criteria may cast doubt on the general applicability of the results [4]. Moreover, a large proportion of unenrolled patients increases study costs and the recruitment time.

The objective of this retrospective observational cohort study was to assess the management and day-90 functional outcomes in comatose patients screened for, but not included in, the HYPERION trial.

## Methods

### Trial design

This was a retrospective study of data collected during patient recruitment for the HYPERION trial, an investigator-initiated, blinded-outcome-assessor, parallel, two-arm, pragmatic, multicenter, randomised controlled trial conducted in 25 intensive care units (ICUs) in France (11 in university and 14 in community hospitals) to compare 33 °C vs. 37 °C after cardiac arrest in a non-shockable rhythm, due to any cause [2]. The trial is described elsewhere [2, 5].

This study was approved by the appropriate French ethics committee (Health Data Hub, N° I02122911192019, approved on 05/05/2020). Survivors screened for, but not included in, the HYPERION trial were informed of the present study; none refused inclusion.

### Patients

We included patients who were screened for the HYPERION trial in 15 of the 25 participating ICUs, but were found to have at least one non-inclusion criterion. All patients admitted after cardiac arrest followed by the return of spontaneous circulation were screened. The HYPERION trial did not include patients with cardiac arrest in shockable rhythm or ICU-admission Glasgow Coma Scale (GCS) scores above 8, as high-quality evidence on outcomes of such patients was available [6, 7]. Also, the HYPERION trial did not include patients younger than 18 years, under guardianship, without health insurance, or for whom informed consent was not obtained.

### Data collection

We reviewed the medical files of each patient. If needed, the day-90 functional outcome was assessed during a telephone call to the patient or family. Investigators were asked to record only the main non-inclusion criterion, as identified based on their clinical acumen.

### Outcome

The primary outcome was the proportion of patients with a favourable functional outcome on day 90, defined as a Cerebral Performance Category (CPC) score of 1 or 2 [8].

### Statistical analysis

Continuous data were expressed as mean ± SD and categorical data as frequencies and percentages. Comparisons used the Chi-square test for categorical variables and Student’s *t*-test or the Mann–Whitney Wilcoxon test, as appropriate, for continuous variables. Differences were considered statistically significant when *P* was less than 0.05. All tests were two-sided. The statistical analysis was performed using STATA version 14.1 (StataCorp, College Station, TX).

## Results

The patient flowchart is Additional file 1: Fig. S1, which shows that 1144 patients were included in the present study. The main non-inclusion criteria were moribund status (*n* = 627), followed by no-flow duration above 10 min (*n* = 506) and logistical reasons (*n* = 284).

Of the 1144 patients, 1130 had day-90 information and, of these, 158 (14%) had good day-90 neurological outcomes (Additional file 1: Fig. S2). The corresponding proportion in the HYPERION trial was 7.9% (10.2% with hypothermia and 5.7% with normothermia). The proportion of patients who received TTM varied across the 15 ICUs participating in this study [from 0/95 (0%) to 31/37 (83.8%) and patients with good day-90 neurological outcomes varied also from 0/34 (0%) to 55/176 (31.3%)]. When we pooled the 1130 patients with day-90 information in this study and the 581 patients included in the analysis of the HYPERION trial, the proportion of patients with good day-90 functional outcomes was 12.1% (204/1711).

Of the 341 patients who received TTM between 32 and 36 °C, 336 had available data on the day-90 outcome. The proportion of patients with good day-90 functional outcomes differed significantly between these 336 patients and the 791 patients who did not receive TTM at 32–36 °C (62/336, 18.5% vs. 94/791, 11.9%, respectively; *P* = 0.003). The most common target temperature was 36° (128/341, 37.5%), followed by 33 °C (106/341, 31.0%), then 34 °C (53/341, 15.5%), 35° (46/341, 13.5%) and 32° (8/341, 2.3%). Table 1 reports the proportions of patients with good functional outcomes according to the HYPERION-trial non-inclusion criterion.

**Table 1:** Number (percentage) of patients with TTM, survival to ICU discharge, and good neurological outcomes (Cerebral Performance Category score 1 or 2) according to presence of non-inclusion criteria for the HYPERION trial in patients comatose after cardiac arrest in non-shockable rhythm, due to any cause

Characteristics	TTM	ICU survival	Good neurological outcome at ICU discharge^a^	Good neurological outcome on day 90^a^
No-flow > 10 min (*n* = 234)	76/234 (32.5%)	10/234 (4.3%)	5/233 (2.1%)	5/233 (2.1%)
Low-flow > 60 min (*n* = 35)	7/35 (20.0%)	2/35 (5.7%)	1/35 (2.8%)	1/35 (2.8%)
Haemodynamic instability (defined as norepinephrine > 1 µg/kg/min) (*n* = 120)	23/120 (19.2%)	21/120 (17.5%)	16/120 (13.3%)	16/120 (13%)
Time from cardiac arrest to screening > 300 min (*n* = 141)	26/140 (18.6%)	48/141 (34.0%)	33/140 (23.6%)	32/140 (22.8%)
Moribund (*n* = 291)	76/291 (26.1%)	10/289 (3.4%)	9/289 (3.1%)	9/289 (3.1%)
Cirrhosis Child–Pugh C (*n* = 7)	3/7 (42.8%)	2/7 (28.6%)	1/6 (16.6%)	1/6 (16.6%)
Pregnant or breastfeeding (*n* = 1)	0/1 (0%)	0/1 (0%)	0/1 (0%)	0/1 (0%)
Inclusion in another study (*n* = 3)	2/3 (66.6%)	1/3 (33.3%)	0/2 (0%)	0/2 (0%)
High risk of bleeding (*n* = 5)	2/5 (40.0%)	1/5 (20.0%)	1/5 (20%)	1/5 (20%)
Logistical reason^b^ (*n* = 307)	126/305 (41.3%)	112/307 (36.5%)	97/303 (32.0%)	93/299 (31.1%)

*TTM* targeted temperature management, *ICU* intensive care unit

^a^The denominator decreased for some variables because data were unavailable in medical charts or patients could not be contacted by phone

^b^Logistical reasons included unavailability of an investigator or of the randomisation software

## Discussion

Patients screened for the HYPERION trial but found to have at least one non-inclusion criterion more often had good day-90 neurological outcomes than did patients included in HYPERION, overall and in both treatment arms. The proportion of non-included patients with good day-90 functional outcomes varied widely across centres, suggesting differences in patient care. For patients managed with TTM, the most common target temperature was 36 °C, followed fairly closely by 33 °C.

When choosing inclusion and non-inclusion criteria for an RCT, a balance should be sought between ensuring external validity by including a large proportion of screened patients and avoiding the inclusion of patients who are unlikely to benefit or likely to experience harm from the trial intervention. The TTM2 trial comparing 33 °C to normothermia with early treatment of fever found no benefits of hypothermia on either all-cause mortality during the trial or functional outcomes [3]. Interestingly, whereas 21% (584/2723) of screened patients were randomised in the HYPERION trial, this proportion was 66% (950/1431) in the TTM1 trial [6] and 44% (1900/4355) in the TTM2 trial [3]. In a prospective study, 30% of ICU patients met the selection criteria for only one of 15 frequently cited RCTs, and 52% met criteria for none of these trials [9].

**Fig. 1:**
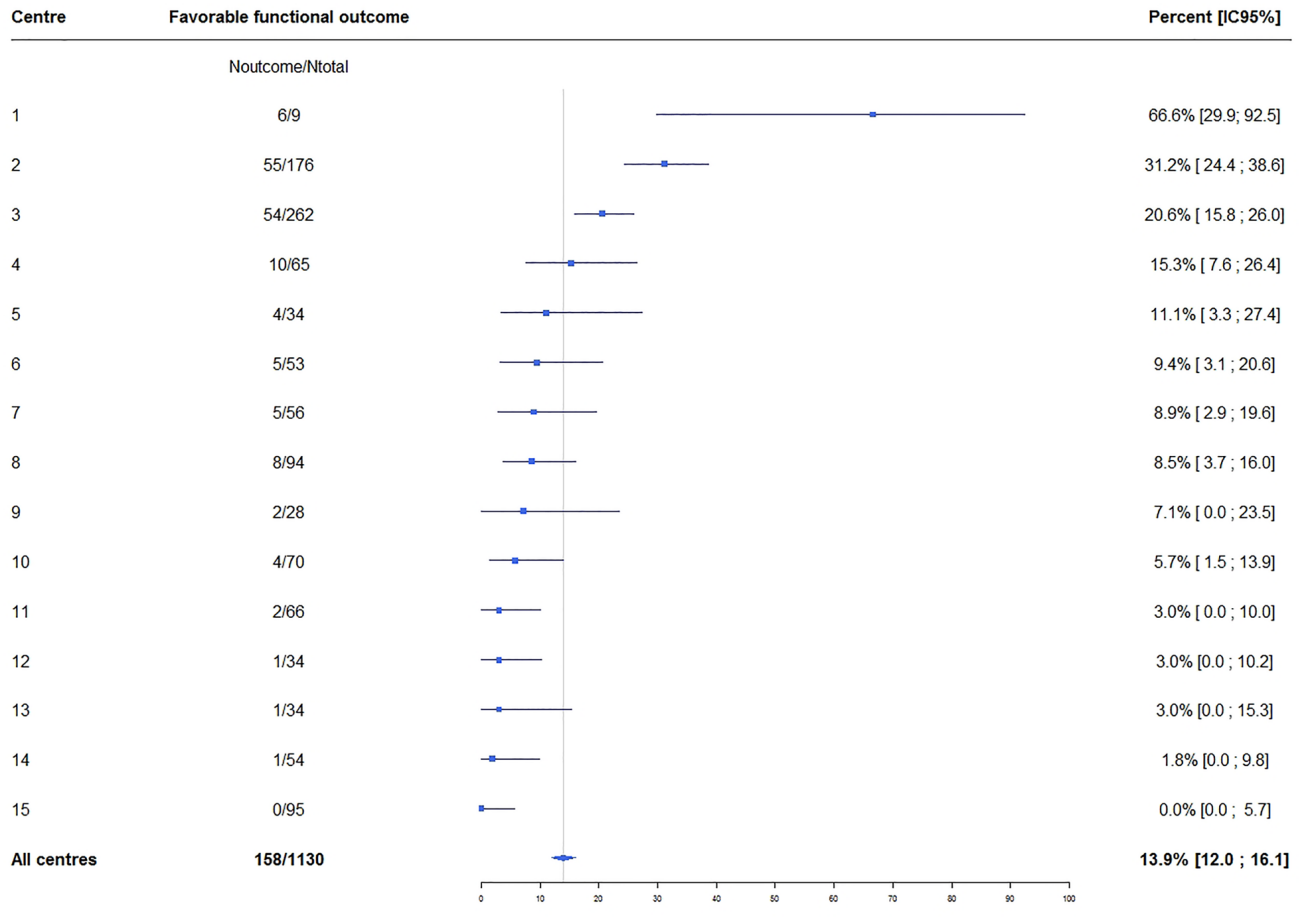
Heterogeneity in functional outcome

Previous data on screening and eligibility for RCTs are scarce. Among critical-care patients, absence of inclusion criteria was more common than presence of non-inclusion criteria [9]. A study reported in 2015 [10] found that about half the patients who were both screened *and* eligible for trials in acute respiratory distress syndrome were not included. Moreover, RCT enrolment was associated with better outcomes compared to those in *eligible* patients who were not enrolled. Interestingly, in patients with acute respiratory distress syndrome, mortality was higher among non-enrolled than enrolled patients, in contradiction to our findings [11]. Despite the generally accepted adverse prognostic significance of most of the non-inclusion criteria used in HYPERION (e.g., moribund status, long no-flow and low-flow durations, and haemodynamic instability), the functional outcomes were better in the non-included than in the included patients. Also, although the frequencies of good neurological outcomes were very low in the groups with long no-flow and low-flow durations (2.1% and 3.1%, respectively), they were not very different from the frequency in the normothermia group of the HYPERION trial (5.7%). A negative self-fulfilling prophecy effect may have occurred, with investigators tending not to include patients whom they expected would die shortly after inclusion. In contrast to this expectation, mortality was lower in non-enrolled patients. Moreover, 3.1% of moribund patients had good day-90 functional outcomes. Outcomes varied across centres, with the proportion of patients having a favourable outcome ranging from 0 to 66% (Fig. 1). The centre with the highest proportion had only 9 patients and the five centres with proportions smaller than 5% had relatively small sample sizes (34 to 66 patients). These variations may be ascribable not only to differences in management, but also to differences in geographic characteristics influencing time to first-responder care and time to admission. Moreover, the selection of centres for invitation to participate in RCTs often depends on network membership, prior collaborations, and personal contacts, which may introduce bias [12, 13].

Third, the use of selection criteria for RCTs limits the general applicability of the findings, which is of great importance. However, selection criteria improve the uniformity of the population, which is essential since a given intervention may benefit some patients but not others. For instance, short-duration intravenous antibiotic therapy is beneficial in patients with septic shock [14], but can be deleterious in those with other presentations [15]. A survey done in the UK assessed the experience of trial recruiters regarding the interpretation and application of eligibility criteria [16]. The main issues reported by the respondents were lack of clarity about what each inclusion and non-inclusion criterion meant, feasibility challenges in assessing the eligibility criteria by obtaining the appropriate investigations within the required timeframe, and uncertainty about whether the criteria were necessary.

The limitations of our study include the retrospective design. Selection and classification bias can occur during medical chart review. More specifically, we were unable to study the subgroup of patients excluded for reasons outside the control of the trial designer. Most of these patients had vulnerability markers such as young age, absence of health insurance, and being under guardianship. Consequently, although the pathophysiology of cardiac arrest is probably similar in this vulnerable subgroup to that in included patients, the treatments and outcomes may differ in ways that might have biased the present study. Although the day-90 functional outcome was usually determined during a telephone interview, this method may have resulted in overestimation of good functional outcomes compared to blinded assessment by a neuropsychologist trained for this specific evaluation. Finally, when comparing patients who did vs. did not receive specific interventions, we were unable to adjust for acute illness severity at ICU admission as assessed by an appropriate score such as the CAHP [17] or OHCA [18], as the data needed to determine these scores were not consistently available in the medical charts.

Further investigations are needed to help translate clinical research findings to the real-life setting. In the specific case of cardiac arrest, whether lowering the body temperature or preventing fever is the most effective intervention should be determined. Finally, given the heterogeneity of cardiac-arrest patients, studies are needed to identify the subgroups most likely to benefit from specific interventions.

## Supplementary Information

**Additional file 1: Figure S1.** Patient flowchart. **Figure S2.** Distribution of Cerebral Performance Category scores on day 90 after screening.
