# Supplementary material for: Insights from patients screened but not randomised in the HYPERION trial
Source: Ann Intensive Care. 2021 Nov 14;11:156. doi: 10.1186/s13613-021-00947-w (PMC8590986; doi:10.1186/s13613-021-00947-w)

**Additional file 1**

**Insights from Patients Screened but Not Randomized in the HYPERION Trial**

***Corresponding author:**

Dr Jean Baptiste LASCARROU, Service de Medecine Intensive Reanimation, CHU Nantes, 44093 Nantes Cedex 1, France

E-mail: [jea](mailto:jean-baptiste.lascarrou@chd-vendee.fr)nbaptiste.lascarrou@chu-nantes.fr

Phone: + 33 240 087 376

Fax: + 33 240 087 377

**Figure S1.** Patient flowchart.

ICU admission after cardiac arrest (n=4466)

♦  Non-inclusion criteria out of the control of the trial designers (n=168):

- *Age <18 years (n=11)*
- *Under guardianship (n=93)*
- *No health insurance (n=19)*
- *Declined to participate (n=45)*

♦  Included in the HYPERION trial (n= 584)

♦  Admitted to any of the 10 non-participating ICUs (n=827)

CPC 1 or 2 on day 90

(n=158/1130, 13.4%)

Received TTM between 32°C and 36°C

(n=341/1141, 29.9%)

♦  Shockable rhythm (n=1435)

♦  Glasgow Coma Scale score >8 (n=308)

Included (n=1144)

ICU admission with GCS score ≤8 after cardiac arrest in non-shockable rhythm (n=2723)

**Figure S2.** Distribution of Cerebral Performance Category scores on day 90 after screening


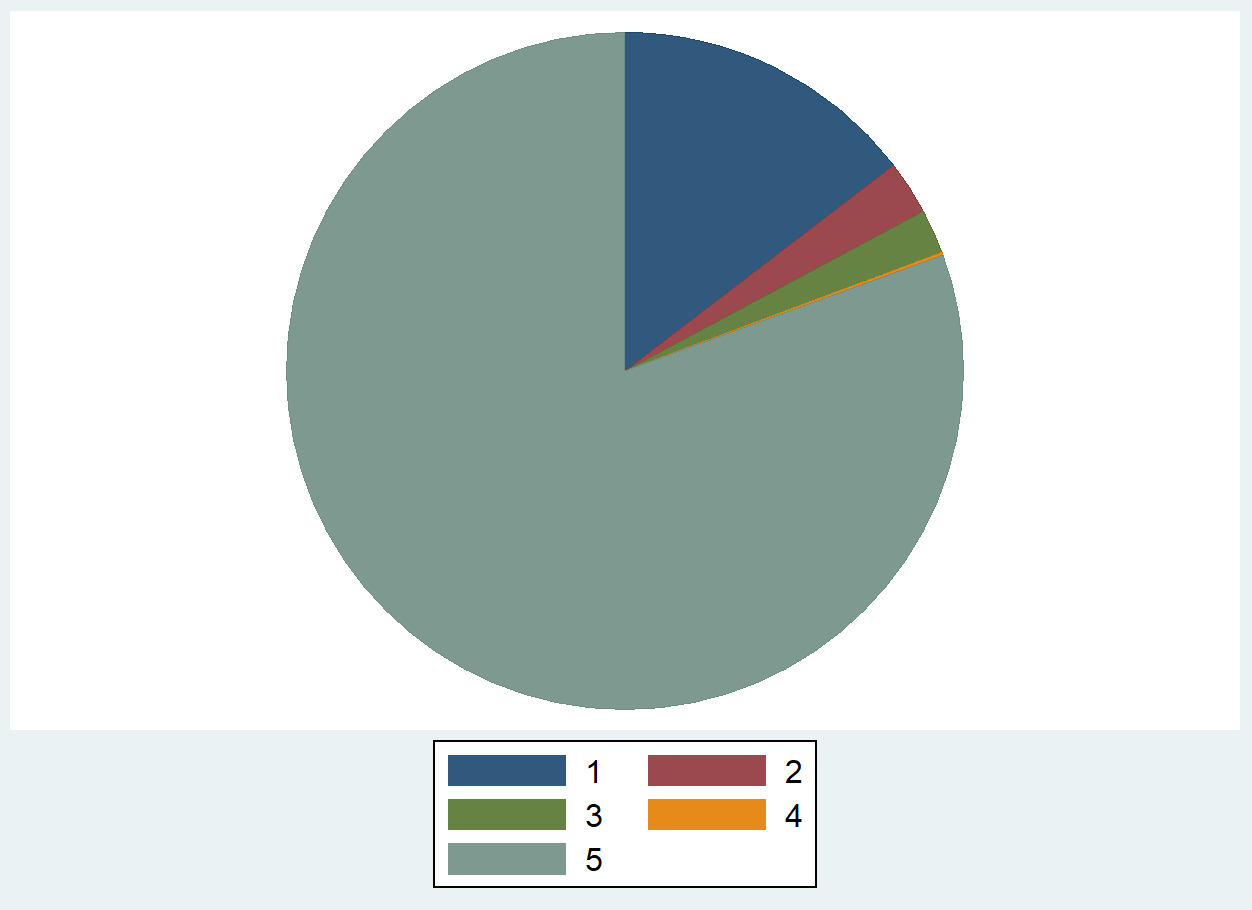

Supplement: Supplementary file 1 — Additional file 1: Figure S1. Patient flowchart. Figure S2. Distribution of Cerebral Performance Category scores on day 90 after screening. [file 13613_2021_947_MOESM1_ESM.docx]
